# Bioremediation of copper in sediments from a constructed wetland ex situ with the novel bacterium *Cupriavidus basilensis* SRS

**DOI:** 10.1038/s41598-022-20930-0

**Published:** 2022-10-21

**Authors:** Alex Kugler, Robin L. Brigmon, Abby Friedman, Fanny M. Coutelot, Shawn W. Polson, John C. Seaman, Waltena Simpson

**Affiliations:** 1 Savannah River National Laboratory, Bldg. 999W, Aiken, SC USA; 2 University of Georgia Savannah River Ecology Laboratory, Aiken, SC USA; 3 Center for Bioinformatics and Computational Biology, University of Delaware, Newark, DE USA; 4 Department of Environmental Engineering and Earth Sciences, Clemson University, Clemson, SC USA; 5 Department of Biological Sciences, South Carolina State University, Orangeburg, SC USA

**Keywords:** Applied microbiology, Environmental sciences, Environmental chemistry

## Abstract

The H-02 constructed wetland was designed to remove metals (primarily copper and zinc) to treat building process water and storm water runoff from multiple sources associated with the Tritium Facility at the DOE-Savannah River Site, Aiken, SC. The concentration of Cu and Zn in the sediments has increased over the lifetime of the wetland and is a concern. A bioremediation option was investigated at the laboratory scale utilizing a newly isolated bacterium of the copper metabolizing genus *Cupriavidus* isolated from Tim’s Branch Creek, a second-order stream that eventually serves as a tributary to the Savannah River, contaminated with uranium and other metals including copper, nickel, and mercury. *Cupriavidus basilensis* SRS is a rod-shaped, gram-negative bacterium which has been shown to have predatory tendencies. The isolate displayed resistance to the antibiotics ofloxacin, tetracycline, ciprofloxacin, select fungi, as well as Cu^2+^ and Zn^2+^. Subsequent ribosomal sequencing demonstrated a 100% confidence for placement in the genus *Cupriavidus* and a 99.014% match to the *C. basilensis* type strain*.* When H-02 wetland samples were inoculated with *Cupriavidus basilensis* SRS samples showed significant (*p* < 0.05) decrease in Cu^2+^ concentrations and variability in Zn^2+^ concentrations. Over the 72-h incubation there were no significant changes in the inoculate densities (10^6^–10^8^ cells/ML) indicating *Cupriavidus basilensis* SRS resiliency in this environment. This research expands our understanding of the *Cupriavidus* genus and demonstrates the potential for *Cupriavidus basilensis* SRS to bioremediate sites impacted with heavy metals, most notably copper.

## Introduction

Copper (Cu), a naturally occurring trace element, can be found throughout the geo- and hydrosphere. Copper is essential for cell function and plays a critical role in various processes such as photosynthesis and cellular respiration^1^. However, high concentrations of Cu are toxic to not only microorganisms, but plants and higher organisms including people. There has been considerable increase in Cu exposure around the world due to anthropogenic activities such as mining, industrial and agriculture applications, and improper waste management^2^. Cu is increasingly applied as an antimicrobial agent in consumer products including construction materials, e.g., roofing shingles and wood for houses and commercial buildings to minimize biodeterioration^3^. Much of the Cu used in consumer products eventually ends up in the environment through disposal and associated waste streams. Copper is difficult to remediate due to its relatively high mobility and ease of transport. This has led to Cu being one of the most common environmental metal pollutants^4^.

**Figure 1:**
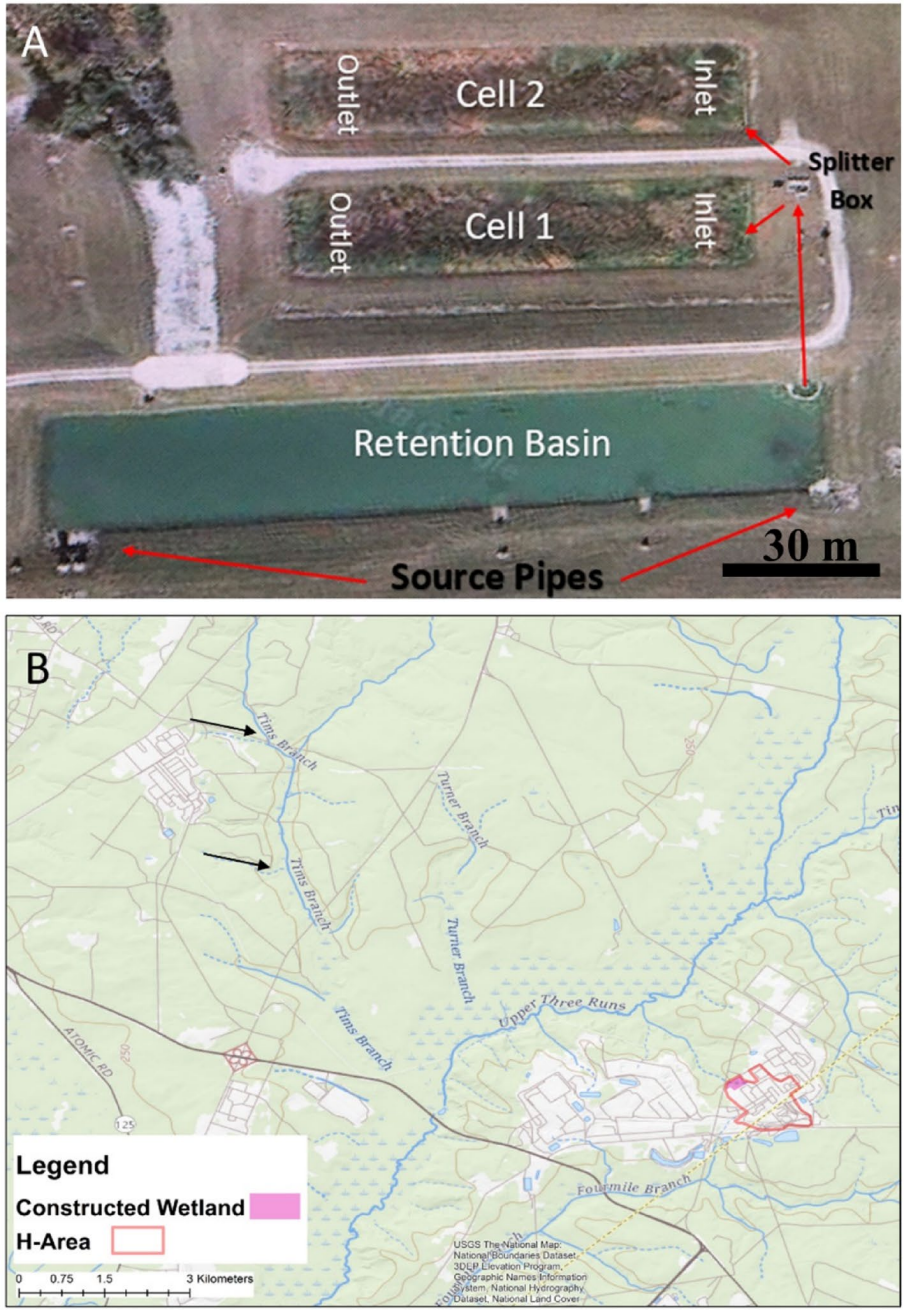
Images of relevant areas of the Savannah River Site. (**A**) GoogleMaps image of the H-02 wetland. Water exits the retention basin through a culvert where it flows to a splitter box that equally feeds into two separate treatment cells. These cells house the water in an artificial wetland environment. Image from *Google Maps*. (**B**) Map showing the Tim’s Branch Creek area where samples from which *Cupriavidus basilensis* SRS was originally isolated. Black arrows highlight likely sites of initial contamination. The box highlights H area and pink box highlights the constructed wetland area, shown in detail in (**A**). Map was created using ArcGIS Destop Release 10 (https://www.arcgis.com/index.html) software by Esri using the USGS National Boundaries Database.

One method of removing Cu from the environment is using constructed wetlands that utilize natural processes associated with vegetation, sediments, and microbial communities to remove contaminants from wastewaters^5^. The H-02 wetland system on the Savannah River Site (SRS) Aiken, SC, (Fig. 1A), was constructed to retain and process water from industrial applications, cooling tower effluent, and surface runoff from processing facilities^6^. This wetland was constructed in 2007 with primary purpose of removing Zn and Cu. It is a free water surface wetland designed to remove heavy metals, specifically Cu and Zn, from the water before it is released to the Upper Three Runs Creek, a regulated stream that empties into the Savannah River. The constructed H-02 wetland mimics the hydrological regime of natural wetlands, with an area of open water, floating vegetation (later referred to as the “organic layer”), and emergent plants^5^. It functions as a land-intensive biological treatment system that is maintained for maximum efficiency^7^. Influent containing elevated metal levels flows through a large area of shallow water where metals and organic matter interact with the surface sediments and/or enter the biogeochemical cycles in the aquatic system.

In a constructed wetland there are four main mechanisms which affect the rates and extent of metal removal: (1) adsorption to fine textured sediments and organic matter, (2) precipitation as insoluble minerals (mainly sulfides and oxyhydroxides), (3) geochemical cycling via plants and bacteria, and (4) deposition of suspended solids^8^. Each of these mechanisms reduces the aqueous concentrations of metal ions by accumulating metals or precipitating them out geochemically; however, typical passive wetland processes do not always reduce metal concentrations to meet regulatory discharge requirements^6^.

One remediation strategy, which is often cost effective and suitable for long term treatment, is bioremediation; a process that makes use of biological systems to catalyze the remediation of contaminants^9^. The application of bacteria can reduce aqueous heavy metals concentrations via metal accumulation through sorption, biofilm accumulation, and uptake^10^. Various microorganisms have a high affinity and tolerance for specific heavy metals^11^. Bioaugmentation, the addition of bacterial cultures to speed up remediation, is often implemented to remove contaminants which can be degraded catabolically ^12^ This method inoculates the wetland with microorganisms with catabolic abilities to enhance bioremoval efficiency. Many studies show that bioaugmentation of constructed wetlands is effective^12–14^. However, applications of bacteria in bioremediation technologies needs to be carefully evaluated as microbial activity has been found to enhance metal mobility, e.g., oxidation of uranium (U), under certain conditions by altering redox state^15^. However, bioremediation strategies such as bioaugmentation, bioleaching, and biostimulation have been applied for Cu removal from soil or to reduce the bioavailability and mobility of target metal ions^9^.

Many microorganisms are difficult to isolate and maintain ex situ, requiring the development of specialized techniques for their growth and cultivation^16^. One such example is *Cupriavidus necator* which is highly resistant to heavy metals and its growth is actively stimulated by Cu^17^. The *Cupriavidus* genus (class β-Proteobacteria, family *Burkholderiaceae*) is described as a group of gram-negative, aerobic rods that are motile by means of petrichous flagella, forming smooth colonies that reach 1–2 mm within 48 h at 30°C^18^. *Cupriavidus* species thrive in metal-rich environments, including Cu-containing soils displaying a mixture of toxic transition metal cations leading to an increased cellular Cu-content. However, the influence of *Cupriavidus* spp. on Cu partitioning in the environment is not well understood. Growth initiation for *Cupriavidus* spp. has been found to be stimulated by Cu^17^. Their ability to take up Cu and prey on other microorganisms is a competitive advantage in the environment^19^. *C. necator* UFLA 01-659 has been previously shown to adsorb all heavy metals tested on the cell wall and membrane, whereas complexation was only observed intracellularly for Cu and Zn^20^.

Metal contamination in the environment is a common problem. One such contaminated area is the Tim’s Branch second-order stream which is a tributary of the Upper Three Runs Creek, which eventually feeds into the Savannah River (Fig. 1B.). From 1954 to 1984 approximately 44,000 kg of depleted uranium and other metals were released into this environment^21^. While there are significant amounts of U, there have also been high levels of other metals reported including nickel (Ni), lead (Pb), and Cu^22^. The high concentrations of metals often taxes the proliferation of many bacterial strains, making them sites of great interest for novel and environmentally relevant specimens^11^.

After the isolation and identification of the novel bacterial species we hypothesized that the novel *Cupriavidus* would have the ability to take up or bind Cu ex situ. In addition, the genera’s natural predatory ability gives it a competitive advantage when bio-augmenting or adding to environmental systems for remediation purposes^19^. For this study, the introduction of newly isolated bacterium was evaluated as a means for enhancing bioremediation in a constructed wetland, especially under conditions where high Cu levels may inhibit indigenous microbial activity. The primary objectives were to isolate a microbe capable of bioremediation of metals commonly associated with the SRS and evaluate its survivability and the efficacy and efficiency of the removal of Cu from impacted wetland sediments.

## Materials and methods

### Isolation, cultivation, and characterization

Initial cultures were isolated from field samples collected from Tim’s Branch Creek a second-order stream on the SRS in Aiken, South Carolina, USA (Fig. 1B)^11^. These samples were collected for a monitoring study of the geochemistry of Tim’s Branch. These samples were plated on tryptic soy agar plates (Fisherbrand™) and placed in a refrigerator at 4 °C. After several days, the plates were observed with clear zones around colonies of what turned out to be the bacterial isolate of interest. After proving to be a Gram Negative (GN) species, the Biolog GN2 kit (Biolog, Hayward, CA, USA) was used for initial identification of the isolated bacterium based on substrate utilization patterns^23^. *B. subtilis* ATCC 49760 was used as a gram positive control.

To isolate the novel strain fresh soil samples were collected from Tims Branch Creek and were initially inoculated onto nutrient agar (Difco, Detroit, MI). Following this the most probable number (MPN) was determined. Cultures were fixed onto glass microscope slides, stained using a gram stain kit (Thermo Fisher Scientific), and observed under an Olympus BX41TF (Tokyo, Japan) microscope.

A further identification of the isolate was confirmed by sequencing of the 16S rRNA gene. DNA was isolated from cultures by placing one isolated colony in 150 µl of QuickExtract Reagent (Epicentre) according to the manufacturer’s instructions. The 16S rRNA gene was PCR amplified (30 cycles: 95C for 45 s, 57C for 45 s, 72C for 120 s) using the 16S-27F (5′-AGA GTT TGA TCM TGG CTC AG; Lane, 1991) and 16S-1492R (5′-GGT TAC CTT GTT ACG ACT T^24^) primers and ExTaq (Takara) polymerase. Sequencing was performed by dye-terminator sequencing on an ABI Prism 3130XL Genetic Analyzer at the University of Delaware Sequencing and Genotyping Center using the 27F, 515F (5′-GTG YCA GCM GCC GCG GTA A^25^), 926R (5′-GGACTACHVGGGTWTCTAAT^25^), and 1492R to obtain bidirectional sequencing of the amplicon.

Resulting sequences were trimmed for quality and to remove primer sequences, then assembled into a 1426 nt contiguous sequence in Geneious (ver 10.2.6). The consensus sequence was analyzed using the RDP Classifier algorithm (ver. 2.1.3; 16S rRNA training set 18^26^ and further homology-based analysis of the sequence was performed using RDP SeqMatch (release 11, update 5; Cole et al. ^27^) and blastn (ver. 2.8.1^28^) against the NCBI nr database (release 242). The sequence and the available near-full length type strain sequences from the genus *Cupriavidus* and *Ralstonia pickettii* were aligned to the SILVA (v.138.1) universal SSU rRNA profile using SINA (ver. 1.2.12^29^). An approximate maximum likelihood tree was constructed with FastTree (ver. 2.1.11^30^) with a GTR + CAT model and optimized gamma likelihoods. Final formatting of the tree was performed in Iroki (rev2020-06-29-1^31^).

### Metal survivability

Metal resistance was tested to determine the viability of the culture to tolerate contaminated sites. Cu and Cobalt (Co) were selected based on previous studies with Cupriavidus^32^. To determine the ability of the novel bacterium to survive in the presence of elevated Cu and Co concentrations eight replicates of each culture treatment condition, including the elevated metal concentrations, were grown in 100 well honeycomb plates compatible with the Bioscreen C turbidity indicator (OY Growth Curves AB Ltd.). The Bioscreen C Automated Microbiology Growth Curve Analysis System allowed direct measures of the novel bacterium growth at varying Cu and Co concentrations (0.0 to 1.5 mM) in R2A (Difco) medium. By measuring the turbidity over time, an optical density (O.D.) curve was generated. The honeycomb plates containing the cultures were incubated for approximately 84 h at 26 °C with medium intensity shaking with growth data collected every hour.

### Antifungal and antibacterial activity

A modified disk diffusion method was used to incorporate the live inoculum. Antifungal activity was tested using the nine fungi listed in Table 1, and three bacterial species were tested *B. subtilis* (ATCC 49760), *B. thuringiensis* (ATCC 35866), and *B. cereus* (ATCC 14579). The test plates were Sabouraud Dextrose Agar (Difco) with varying concentrations of Cu (0, 0.1, 0.35, 0.75, and 1.5 mM) which had been inoculated with the fungi and bacteria described above. The microbe of interest was grown on a sterile Whatman 40 (12.5 cm) filter paper which had been placed on an 2A agar plate and allowed to grow for 48 h before being transferred to the test plates. Sterile tweezers were used to transfer both sterile and inoculated filters to each of the test plates which were then sealed with parafilm and incubated at room temperature for 72 h and checked daily for growth and appearance of clear zones which would indicate microbial inhibition.

**Table 1:** Mold spp. tested for sensitivity to *Cupriavidus basilensis SRS.*

Molds identification	Clear zone developed?
*Pithomyces spp.*	Yes
*Pseudallescheria-boydii*	Yes
*Myrothecium verrucaria*	Yes
*Exophiala-moniliae*	Yes
*Scytalidium spp.*	No
*Alternaria-species*	Yes
*Penicillium-species-GC* subgroup A	Yes
*Phoma spp.*	No
*Myrothecium verrucaria*	Yes

### Antibiotic resistance

To determine antibiotic resistance fresh bacterial stocks were inoculated into 5 mL R2A (pH 7) and incubated at 30 °C with shaking (100 rpm) for 48–72 h. Cultures were serially diluted, and aliquots (250 µL) were plated onto R2A agar incubated at room temperature. ETEST strips consisting of a predefined gradient of antibiotic concentrations on a plastic strip were used to determine the Minimum Inhibitory Concentration (MIC) of antibiotics (BioMérieux). The sensitivity ranges for the five antibiotics tested are 0.016–256 µg/mL for tetracycline and ampicillin and 0.005–32 µg/mL for ofloxacin, cefotaxime, and ciprofloxacin. The strips were applied in triplicate to pre-inoculated R2A agar plates. Plates were incubated at room temperature for 7 days, and MIC determined at 2 and 7 days. Sensitivity and resistance were determined based on the MIC as determined by the extent of plate clear zones.

### Constructed wetland parameters

The H-02 wetland is an operational engineered wetland that was constructed between 2006 and 2007 to utilize natural processes associated with natural and amended sediments, plants, and microbial communities to remove contaminants from building and run off waste waters at the SRS (Fig. 1). The water comes from industrial applications, including building process water, cooling tower effluent, and surface runoff from several processing facilities (Fig. 1). The wetland was constructed using a geo-synthetic impermeable liner and is amended with gypsum to prevent elevated levels of Cu and Zn, the site-specific metals of interest, from entering the Upper Three Runs Creek which eventually feeds into the Savannah River. The removal process makes use of the sulfur geochemistry cycle precipitating copper and zinc as sulfide minerals^33^. Figure 1 illustrates where the water exits the retention basin through a culvert and flows to a splitter box that equally feeds into two separate 0.25-ha treatment cells, which discharge to the stream (Fig. 1A). Previous studies by the Savannah River Ecological Laboratory (SREL) which currently manages the wetland reported 23 total species of vascular aquatic plants and 24 species of amphibians and reptiles^34^. Further in depth and detailed descriptions of the free-surface wetland system can be found in Xu, et al. ^35^.

### Microcosm experiment utilizing constructed wetland samples

Water, sediment, and organic layer (OL) samples were collected at the inlet and outlet of the wetland, and water samples were also collected from the retention basin, in triplicate. Samples were placed on ice and processed within 24 h for experimental treatment. The sampling was conducted in different locations to collect a wide a range of Cu and Zn concentrations and speciation. For bioaugmentation testing the isolate was cultured in liquid R2A medium for 5 days at 25 °C, and 80 rpm on a rotary shaker. Bacteria were harvested via centrifugation, the supernatant discarded, and the bacterial pellets washed 2X with sterile phosphate buffered saline (A Buffer, Difco, Detroit MI).

To eliminate the native bacterial community impact, inlet and outlet water samples were autoclaved before inoculation with the isolate. A subset of the water basin samples was used as negative control with no inoculate added, which would demonstrate any effects of native sediment microbiota. Initial wetland samples were inoculated with the isolate by adding 1 mL of vortexed bacterial suspension in 50 mL tubes containing water, sediment or organic layer samples. All tests were performed in triplicate and were cultured on an orbital shaker for 72 h (Day 3) at room temperature.

On day 0 and Day 3, water, sediment, and organic layer samples were centrifuged, the supernatant collected, filtered (nylon syringe filter 0.22 µm pore size) and acidified (2% final concentration HNO_3_) prior to analysis for Cu and Zn by inductively coupled plasma mass spectrometry (ICP-MS; NexION 300X, Perkin-Elmer Corp.) according to the QA/QC protocols outlined in EPA Method 6020B. The pH of the microcosms was monitored at 24 h, 48 h and 72 h using a Seven Excellence pH meter (Mettler Toledo) equipped with a InLab Micro Pro-ISM pH probe (P/N: 51344163). The pH and dissolved Cu and Zn concentrations at T0 (before inoculation) vs T3 (three days post-inoculation) were compared using analysis of variance (ANOVA, aov function) and the emmeans package 1.2.1 for R.

Microcosm microbial densities as determined via colony forming units (CFUs) were determined on R2A plates with 1 mM Cu to select for the isolate. Bacteria densities were determined by serially diluting 1-mL aliquots of the samples in sterile PBS, spreading the suspension (0.1 mL) on R2A medium with 0.1 mM Cu to select for the isolate, and incubating for one week at room temperature. The initial bacterial concentration was determined by placing 0.1 mL of culture on an R2A plate and performing a plate count, without Cu as a selective pressure. On Day 3, a subsample of the inoculated sediment, organic layer and water samples were used determine the isolate’s growth.

### Statistical analysis and modeling

Software R (https://www.r-project.org/) was used for all statistical analyses. All experiments were performed in triplicate. Differences between groups were determined using the ANOVA “aov” function followed by emmeans for post hoc comparison between groups. R version 3.4.4 and RStudio 1.1.442 were used.

The Eh–Ph diagram was created using Geochemist’s Workbench ® (GWB v 9.0)^36^.

## Results

### Isolation, cultivation, and characterization

**Figure 2:**
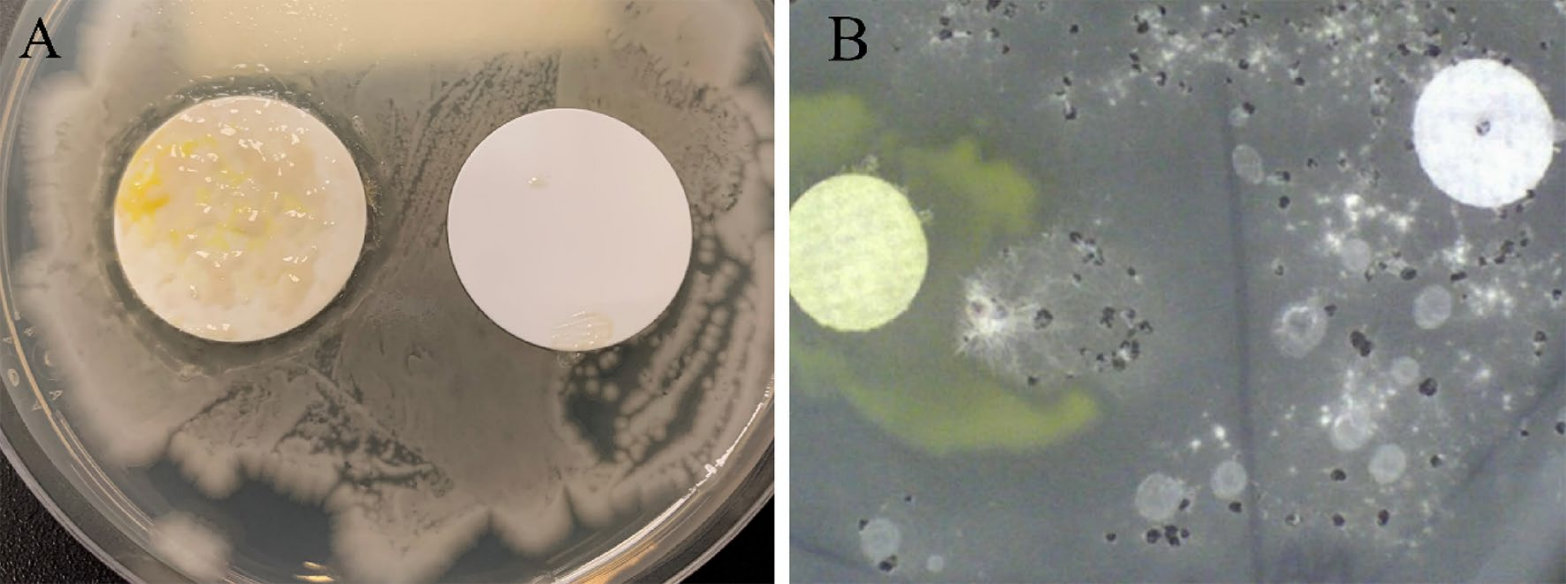
Plates of *Bacillus subtilis* (**A**) and *Exophiala-moniliae* (**B**) were grown on plates of R2A agar, had filters (25 mm) overgrown with *Cupriavidus basilensis* SRS placed on them and were incubated. Clear zones developed around *Cupriavidus basilensis* SRS containing filter after incubation, and no zone developed around the blank control. The right white filter (**B**) contained no *Cupriavidus basilensis* SRS*.*

Initial culture plates were shown to have clear zones which appeared to mimic antimicrobial properties as noted by the ability to create clear zones in the presence of environmental microorganisms, including both fungi and other bacteria (Fig. 2). The yellowish colony was streaked onto R2A agar plates (Difco catalog no. 218263), an undefined low-nutrient medium and grew. Isolated cultures were further characterized on Biolog GN with results demonstrating a probability of 0.660 match to *C. necator* (Table 2) at 24 h. Biolog identifications are accepted as correct if the similarity index of the genus and species name is 0.750 or greater at 4 h or 0.500 or greater at 24 h incubation^37^. The next three closest matches were all closely related *Cupriavidus* species: *C. campiensis*, *C. gilardii*, and *C. pauculus*.

**Table 2:** Biolog results for *Cupriavidus basilensis SRS* characterization*.*

Species ID: *Cupriavidus necator*					
	SIM	DIST	PROB	Organism type	Species
1	0.660	4.914	0.660	GN-NEnt	*Cupriavidus necator*
2	0.196	5.374	0.311	GN-NEnt	*Cupriavidus campiensis*
3	0.014	6.960	0.026	GN-NEnt	*Cupriavidus gilardii*
4	0.014	6.960	0.026	GN-NEnt	*Cupriavidus pauculus*

**Figure 3:**
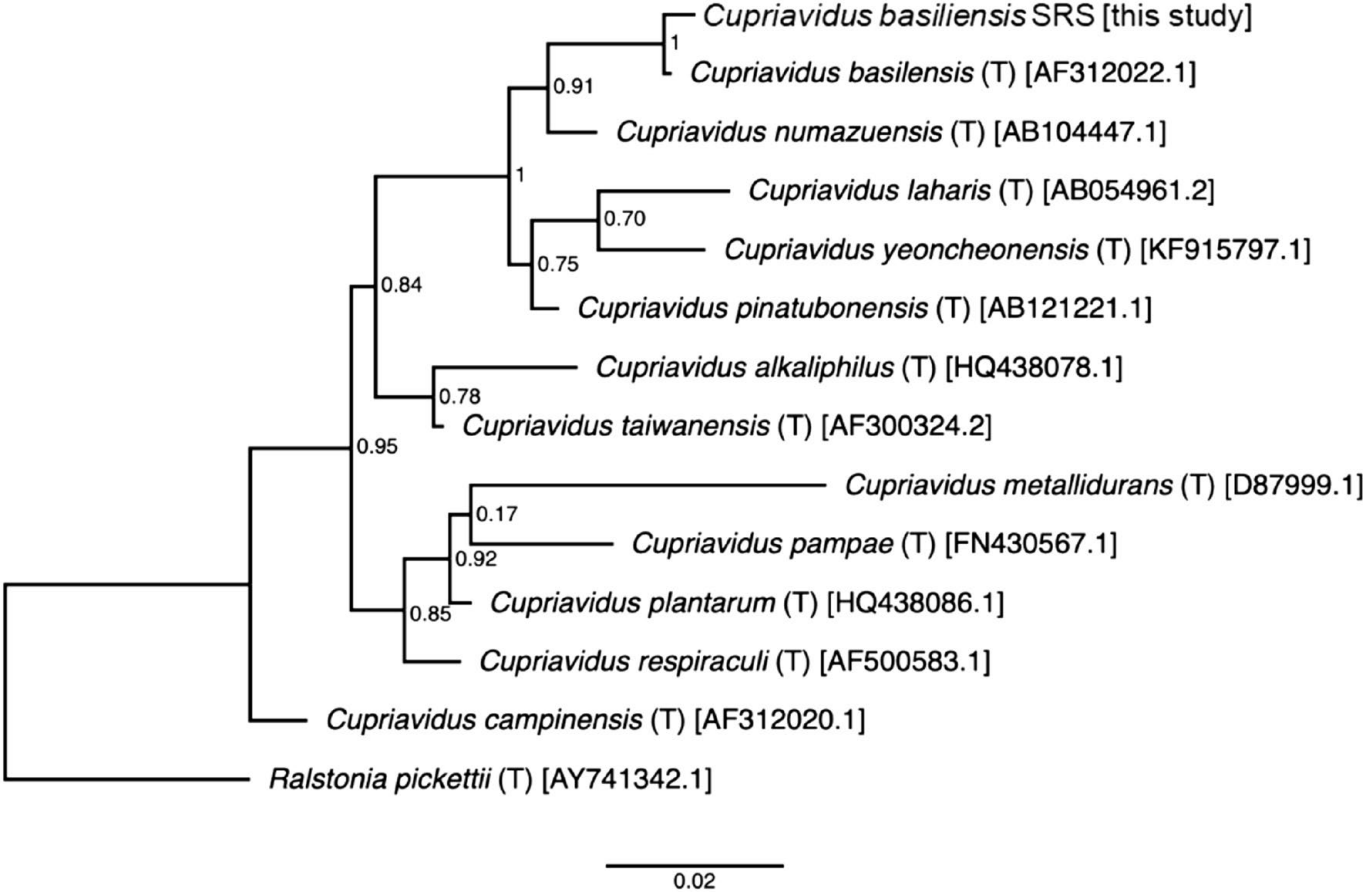
Phylogenetic tree of 16S rRNA relating *Cupriavidus basilensis* SRS to other closely aligned *Cupriavidus* spp, resulting from a near-full length nt sequence indicating a near 100% confidence that the *Cupriavidus basilensis* SRS belongs to the *Cupriavidus* genus.

The 16S rRNA gene was sequenced from the isolate, producing a near-full length 1426 nt sequence. The sequence analysis indicated 100% confidence placement in the genus *Cupriavidus*. The RDP SeqMatch tool and BLASTn against the NCBI non-redundant sequence database indicated exact matches to numerous environmental isolates in the genus *Cupriavidus*, with the closest characterized type strain, *Cupriavidus basilensis* (DSM 11853; AF312022) having a 99.014% nucleotide sequence identity. An approximate maximum likelihood phylogenetic tree agrees with taxonomic placement of the isolate in the genus *Cupriavidus*, with the *C. basilensis* type strain as the closest neighbor (Fig. 3). The bacterial isolate is referred to as *Cupriavidus basilensis* SRS*.*

### Metal and antibiotic survivability

**Figure 4:**
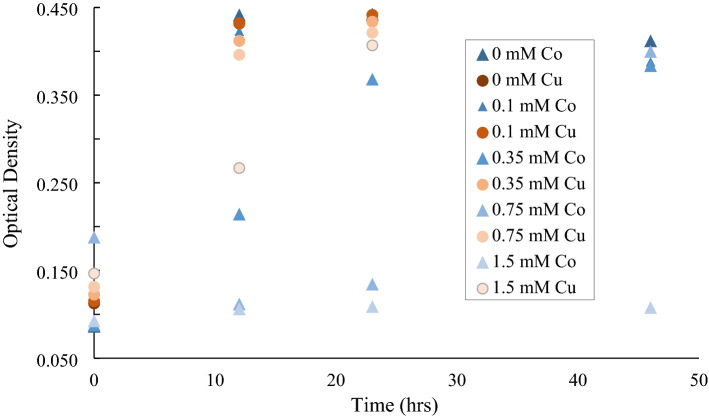
Growth of *Cupriavidus basilensis* SRS with Cu and Co in liquid medium with Bioscreen for 23 h and 46 h for Cu and Co, respectively. Five different concentrations were monitored, 0, 0.1, 0.35, 0.75, and 1.5 mM. Growth was monitored via optical density at 600 nm. Average of 5 runs, error bars are smaller than symbols shown. The general trend shows a decease as metal concentrations increase; however, Co is significantly more inhibitory than Cu.

Based on the fact the isolate came from soils at a heavy metal contaminated site, testing was performed to examine the isolate’s resistance to Co and Cu (0.1–1.5 mM). Results showed that there was *Cupriavidus basilensis* SRS growth at all concentrations (0.0–1.5 mM) for Cu (Fig. 4). However, in this range for Co, *Cupriavidus basilensis* SRS showed a longer lag time for growth above 0.1 mM and growth appeared inhibited at the higher concentrations. (Fig. 4). Longer growth times were observed for *Cupriavidus basilensis* SRS at 0.75- and 1.5 mM Cu, with some reduction in gr (Fig. 4).

For *B. subtilis*, *B. thuringiensis*, *B. cereus* spread plates there was a zone of inhibition around *Cupriavidus basilensis* SRS colonies (Fig. 2A). All the zones were similar in size. This was not dependent on the copper concentration as the *Cupriavidus basilensis* SRS showed the same zone size as ranging from 0 to 1.5 mM Cu concentrations and *Cupriavidus basilensis* SRS on the filter paper hole punched dots. As an example, *B. subtilis* spread plate showing similar zones of inhibition in the presence of *Cupriavidus basilensis* SRS inoculated nitrocellulose filters with differing levels of Cu concentrations 0.75 mM and 1.0 mM, is provided in Fig. 2A.

In addition to the ability of *Cupriavidus basilensis* SRS to inhibit the growth of other microorganisms, the resistance to several antibiotic effects were examined. Of the five antibiotics tested, *Cupriavidus basilensis* SRS was resistant to three, Tetracycline (0.75 µg/mL), Ciprofloxacin (0.50 µg/mL), and Floxacin (0.75 µg/mL). No resistance was observed with Ampicillin or Cefotaxime. The minimum inhibitory concentration (MIC_90_) for *Cupriavidus* species reported elsewhere for ciprofloxacin is 1 mg/L and the reported breakpoint for resistance is 18^38^.

### Antifungal and antibacterial activity

To further characterize the antimicrobial capabilities of this bacterium to generate antimicrobial clear zones was investigated. Results from testing *Cupriavidus basilensis* SRS for inhibition of nine fungi on agar plates are shown in Table 1. Of the nine fungi tested, seven were sensitive to *Cupriavidus basilensis* SRS growth and *Phoma* and *Scytalidium spp.* were not affected. Of the nine fungi tested, seven were sensitive to *Cupriavidus basilensis* SRS growth while *Phoma* and *Scytalidium spp*. were not affected. Clear zones around *Cupriavidus basilensis* SRS inoculated filter paper circles w/0.35 mmol Cu (left) and the control with no Cu (right) or *Cupriavidus basilensis* SRS on a plate covered with *Exophiala-moniliae* are shown in Fig. 2B. The clear zone between *Cupriavidus basilensis* SRS and the test microorganisms indicates the inhibition is caused by secreted extracellular factors. This antibacterial activity was present regardless of copper concentration as show by the antimicrobial clear zones in Fig. 2A.

### Bioaugmentation of metal contaminated wetland sediments

**Figure 5:**
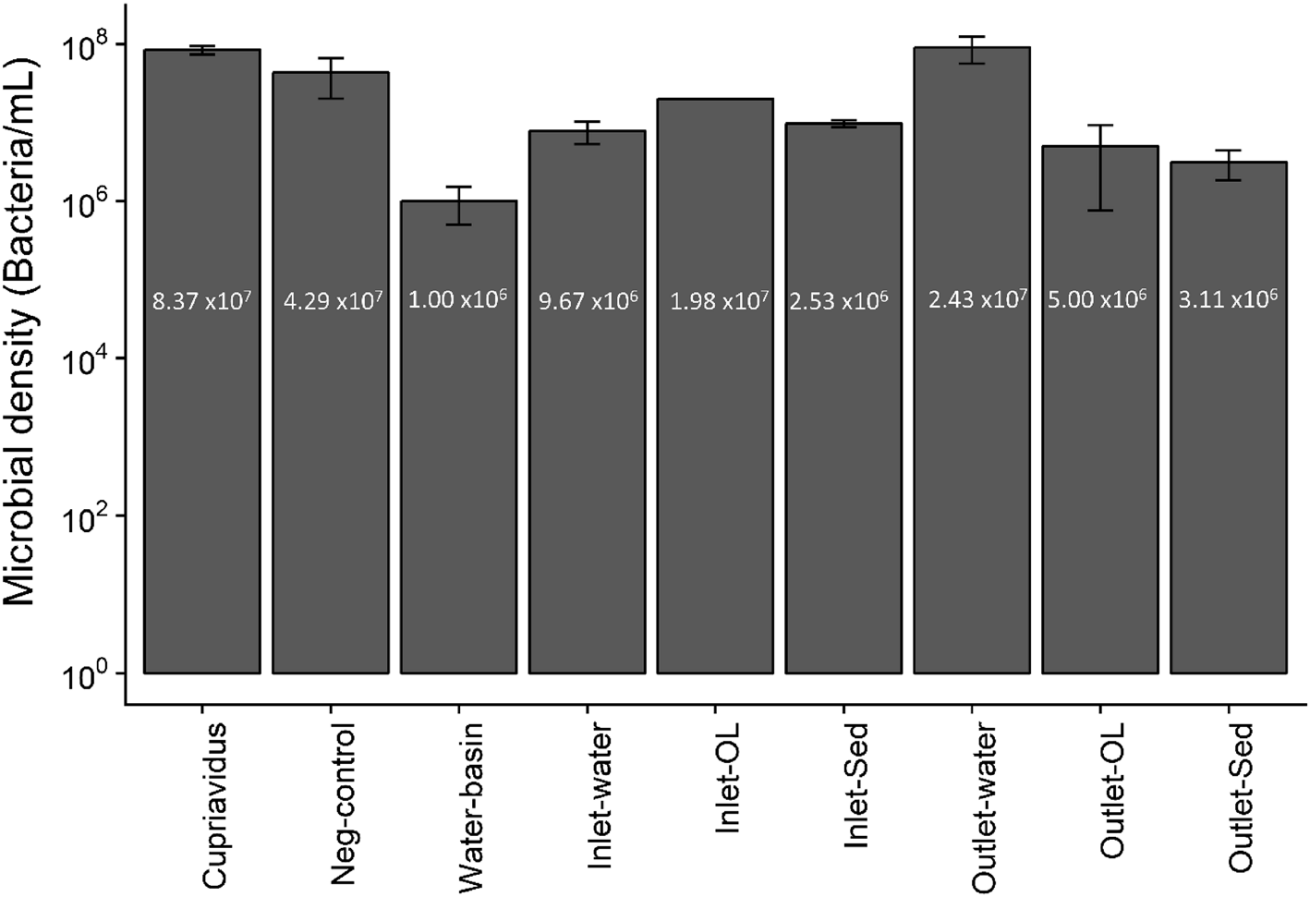
Microbial density of *Cupriavidus basilensis* SRS in various wetland test samples after inoculation. *Cupriavidus* acts as a control sample for comparison, and denotes the initial microbial concentration added to the wetland samples. The data is shown as mean values with the error bars representing the 95 percent confidence intervals around the average value. Samples measured in triplicate.

Figure 5 shows the initial microbial density of the microbial stock, negative controls (wetland water with no bacteria added), and post inoculation of wetland samples, in triplicate, as measured by CFUs after three days of incubation. Densities in the negative controls indicate Cu tolerant bacteria in the water as the agar plates contained 1.0 mM Cu, however a Welch’s two sample t-test compared dissolved Cu and Zn concentrations in autoclaved and non-autoclaved samples, to examine the effect of indigenous microorganism in response to Cu. There was no significant difference in dissolved Cu and Zn concentrations between sterilized via autoclaving, and non-sterilized samples (*p* >  > 0.05).

Microbial densities in wetland samples ranged from 10^7^ to 10^9^ cells per mL (v/v) and did not vary significantly in the wetland samples (Fig. 5), and the outlet water microbial densities also did not vary significantly. This indicates that the inoculation of the bioaugmenting microbe was a success over this 72-h period. The concentration of copper tolerant bacteria, which was negligible in uninoculated samples, indicates that any the metal concentrations in the experiment result from the direct application of the *Cupriavidus basilensis* SRS *.*

While there were trace Cu tolerant bacteria in the water as demonstrated by the negative controls (Fig. 5), these results suggest that the indigenous microbial community does not affect dissolved Cu and Zn levels. The initial pH and dissolved Cu and Zn concentrations vs T3 (three days post-inoculation) were compared using analysis of variance (ANOVA, aov function) and the emmeans package 1.2.1 for R (R version 3.4.4 and RStudio 1.1.442).

**Figure 6:**
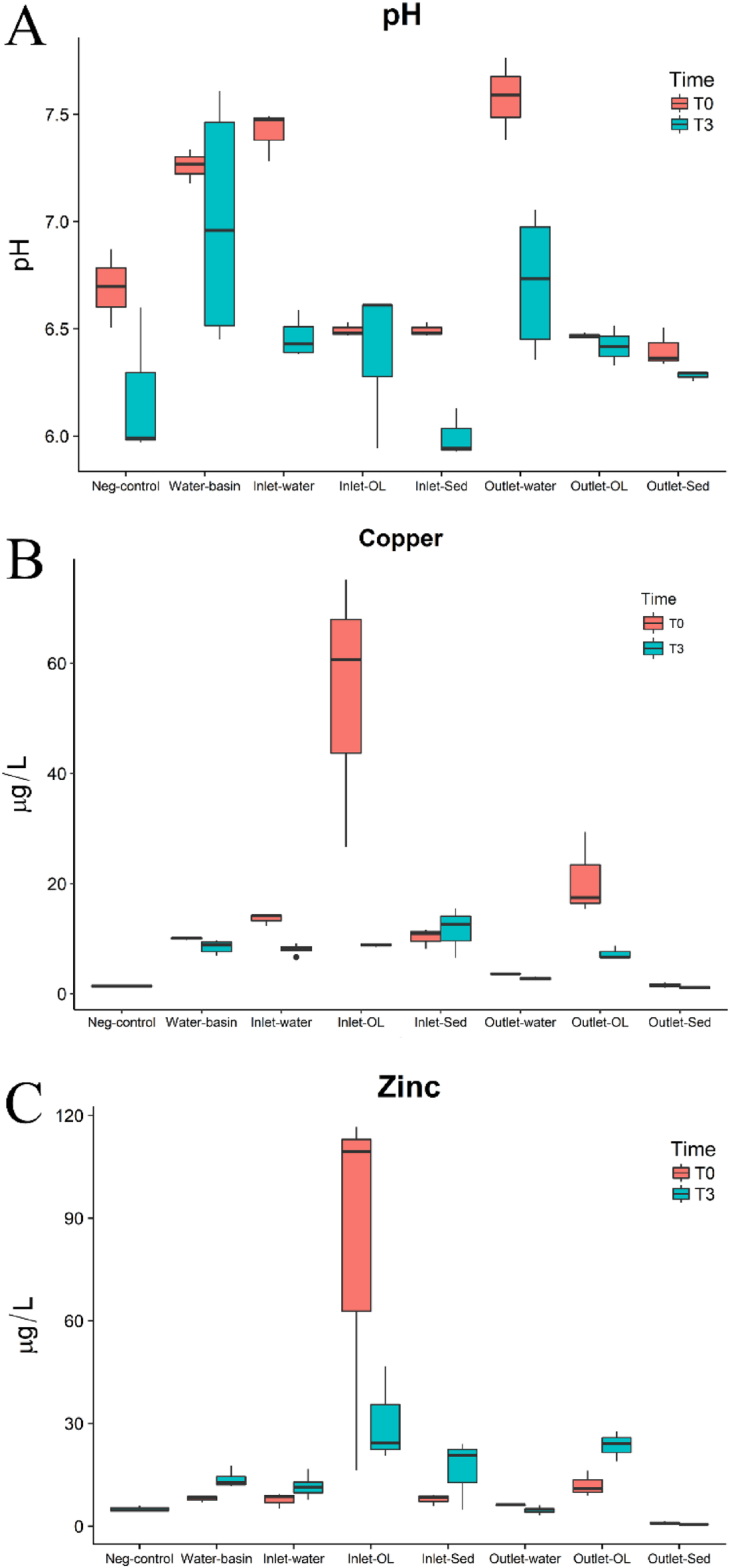
Changes in water chemistry after incubation with *Cupriavidus basilensis* SRS after three hours, depicted with boxplots. The pH of the water, sediment (sed) and organic layer (OL) sample treatments are shown in (**A**); Cu ion concentrations in the water, sediment (sed) and organic layer (OL) in (**B**); and Zn ion concentrations in the water, sediment (sed) and organic layer (OL) in (**C**). The data is shown as mean values with the error bars representing the 95 percent confidence intervals around the average value; samples measured in triplicate.

The pH of all microcosms generally decreased over the course of the microbial exposure (Fig. 6). The inlet and outlet water pH values were found to vary significantly over the course of the microcosm testing *p* < 0.001 and *p* < 0.005 respectively. Figure 6A shows the pH variation as a boxplot for various samples at T0 and T3. The pH of the solution varies between the samples and decreases significantly lower for the inlet and outlet water samples after inoculation at T3. Statistical analysis confirmed these results, finding that water samples from the wetland inlet and outlet are different after inoculation (with *p* = 0.005 and *p* = 0.0025, respectively).

Figure 6B shows the dissolved Cu concentrations for all samples before (T0) and after (T3) inoculation with *Cupriavidus basilensis* SRS. The dissolved Cu concentrations show generally a decreasing trend after inoculation but is not significantly different for all water and sediment sample class. It does appear quite different for organic layer sample treatment (i.e., Inlet OL and Outlet OL) for T0 vs T3, with the most dramatic results evident in the Inlet OL (all associated *p* values < 0.05). Figure 6C shows the dissolved Zn concentrations for all sample classes before and after inoculation. In general, the dissolved Zn concentration tends to increase somewhat after inoculation with *Cupriavidus basilensis* SRS, except for Inlet organic layer samples, which decreased. The statistical results confirm a significant difference between T0 and T3 for Zn Inlet OL (*p* = 0.0041) samples but no difference for all the other samples when inoculated.

## Discussion

### Isolation, cultivation, and characterization

The initial observed clear zones around identical cultures in environmental samples showing competitive antimicrobial activity gave an indication that *Cupriavidus basilensis* SRS was of interest. Samples were identified as a gram-negative rod-shaped bacterium intially identified as a *Cupriavidus* by a "fingerprint" of carbon sources used for identification (Table 2). Previous phylogenetic analyses of metal tolerant Gram-negative microbial isolates from this SRS site based on partial 16S rRNA sequences, showed similarity to *Cupriavidus metallidurans* CH34^11^. As shown in Fig. 2, *C. basilensis* was the closest species-level match (99.014%) to *Cupriavidus basilensis* SRS based on the 16S RNA nucleotide sequence analysis. *Cupriavidus* spp. has been found to have catabolic potential for degrading aromatic compounds and persists under heavy metal stress conditions facilitated by genes encoding heavy metal transport/detoxification proteins^39^. However, *C. basilensis* has not been reported to be antimicrobial like the *Cupriavidus basilensis* SRS isolate^40^, indicating a distinctive niche for this microorganism. Based on these results, we propose that *Cupriavidus basilensis* SRS is its own strain, distinct from other *Cupriavidus*. It has been demonstrated that long term exposure to copper in sediments causes a decrease in overall biomass and biodiversity of both bacterial and fungal samples when analyzing samples via RNA reverse transcription^41^. This data supports the earlier work done using DNA studies^42^, and due to the sensitivity of many organisms to copper is the likely cause of the proliferation of this particular species. Additionally, heavy metal ions coregulate many genes for antibiotic resistance which decreases overall antibiotic susceptibility^43^. *Cupriavidus basilensis* SRS showed resistance to three of the five tested antibiotics, ofloxacin, tetracycline, and ciprofloxacin, which follows the already accepted trends in the literature.

### Metal survivability

Based on the fact the isolate came from soils at a heavy metal contaminated site, testing was performed to examine the isolate’s resistance to Co and Cu (0.1–1.5 mM). Although the mechanism of Cu uptake by *Cupriavidus basilensis* SRS is unknown, the isolate has the potential for the remediation of metal contaminated waters or soils. Operons responsible for metal transport have also been observed in other bacteria, including *Cupriavidus gilardii*^26^. *C. gilardii* CR3, a species resistant to multiple metals, was demonstrated to have operons responsible for efflux pumps, for cadmium (Cd), Zn, Cu, and gold (Au) linked to heavy metal resistance through sequencing analysis. Vicentin, et al. ^20^ found that the distribution of Cu and Zn within the cells of *Cupriavidus* spp. UFLA 01-659 *C. necator* proves capacity for metal removal by ion efflux pumps. The species then complexes metals, forming intracellular deposits. It has also been shown that *Cupriavidus metallidurans* CH34 is capable of exporting Cu^+^ from the cytoplasm by the Cu-efflux pump CupA and has the periplasmic Cu(I)-oxidase CopA gene ^44^. The CopA gene has been linked to copper resistance in various other microorganisms including *e. coli*^45^. CH34 has also been shown to produce a periplasmic protein CzcE which in the oxidized state four Cu(II) atoms but in the reduced state has four Cu(I) atoms. When paired with the pump system this may prove to be an effective means of removing solubilized Cu^46^. This pathway would oxidize the soluble Cu^2+^ which would reduce the concentration of solubilized copper corresponding to the trend seen in Fig. 6. The fluctuations in Zn may be due to release from sediments during incubation. Zn has been demonstrated to involve ion exchange and/or compensation of negative charges at the surface of wetland substrates revealing that Zn is potentially desorbable from the substrate while Cu and Pb removal efficiencies are above 90%^47^.

**Figure 7:**
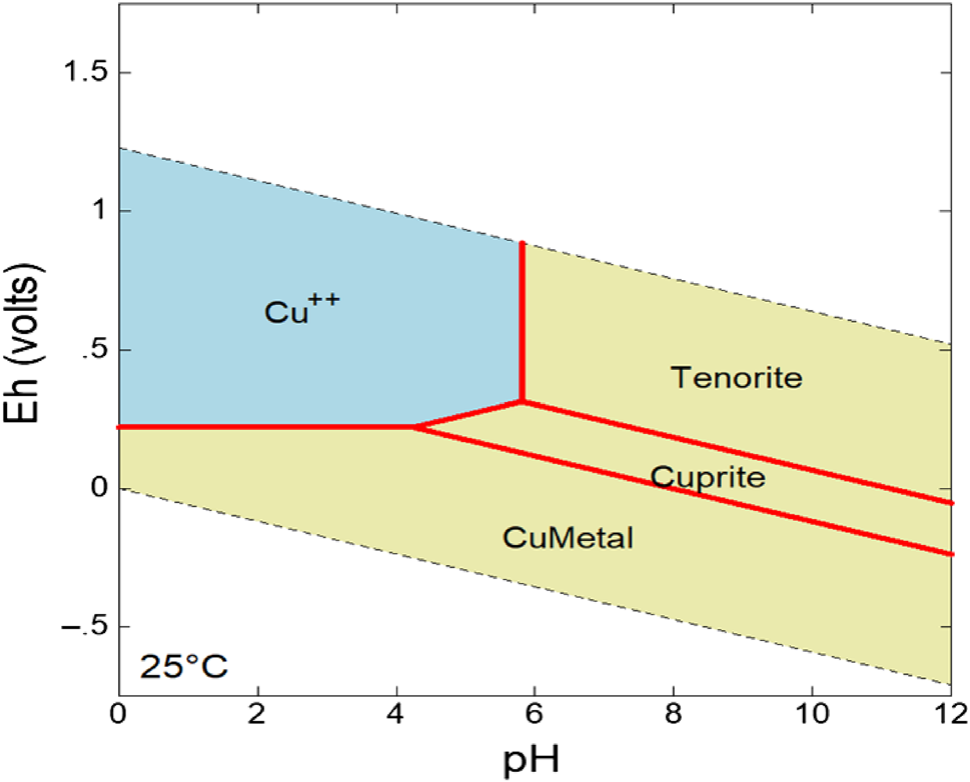
Model for microbially induced changes from pH 6 to 9 over three days for Cu^+2^. Model reflects standard temperature and pressure.

Gram-negative bacteria are susceptible to the accumulation of metals within the cytoplasmic and periplasmic spaces, such as Cu^48^. Previous evidence that Cu-resistant bacteria can respond very rapidly to competition, makes them useful potential tools for bioremediation^49^. *Cupriavidus basilensis* SRS also appears competitive with other bacteria and fungi (Fig. 4; Table 1) and the densities observed indicated it was thriving in the wetland water ex situ (Fig. 5). Microbial densities in the wetland samples ranged from 10^6^ to 10^9^ cells per mL, indicating survival and growth in an environment with elevated levels of Cu and Zn as well as possibly contributing to lower dissolved Cu (Figs. 6 and 7). Nevertheless, the microbial densities remained relatively high (Fig. 5) given the metal concentrations in those samples. A longer incubation may prove more effective in to create a more resilient growth or biofilm. This suggests that while *Cupriavidus basilensis* SRS may inhibit growth of microbes in a laboratory environment in an active remediation environment the overall impact on microbial density and diversity would be limited, and possibly nonexistent.

#### Antifungal and antibacterial activity

Previous bacteria have been shown to have antifungal properties^50,51^. Gram-negative bacteria have been shown to limit or eliminate fungal growth through various means including extracellular lytic enzymes, salicylic acid, antibiotics, and the production of volatile metabolites^52–54^. The antifungal activity of *Cupriavidus basilensis* SRS (Table 1) highlights the uniqueness of this bacterium compared to other members of the genus, who as far as authors are aware, have not been demonstrated to be antifungal to any significant degree. The clear zone between *Cupriavidus basilensis* SRS and the test microorganisms (Fig. 2B) indicates the inhibition is caused by secreted extracellular factors. Additionally, *Cupriavidus basilensis* SRS inhibited the growth of various other microorganisms, which may have been due to non-obligate bacterial predation which has been reported in *C. necator*. *Cupriavidus necator* has been confirmed to predate on *b. subtilis*, and that this killing was mediated via extracellular factors, and that a quorum of predatory cells was not required^55^. Due to the phylogenic closeness of C. *necator* to *Cupriavidus basilensis* SRS it is likely that similar extracellular factors mediate the bactericidal nature of the species, as demonstrated by the clear zone in Fig. 2B. This relationship with other bacteria suggests that even in resource limited environments *Cupriavidus basilensis* SRS will be able to reduce the Cu contamination.

#### Bioaugmentation of metal contaminated wetland sediments

As shown on Fig. 6B, the organic layer samples (inlet and outlet) showed a significant decrease in dissolved Cu concentration but in the water and sediment samples, the dissolved Cu concentration tended to decrease but it is not statistically significant. Even so, the trend suggests that the addition of *Cupriavidus basilensis* SRS to the wetland samples can potentially reduce the dissolved Cu concentrations. As shown by Das, et al.^56^, soil samples exposed to *Cupriavidus spp* results in a significant decrease of Cu concentration in 10 days.

Wetland interactions of plants, microbial activities, sediments, and water geochemistry can alter redox conditions, pH and organic matter and influence the chemical speciation and mobility of metals, including Cu^57^. These interactions can include Cu binding to inorganic material through chemical complexation. Potential Cu biosorption by biomass is possible by the non-specific binding or physical–chemical complexation reactions between dissolved metal species and charged biofilm or cell surface-associated or extracellular polysaccharides and proteins^58^. Metal uptake in wetlands by both biotic and abiotic means can also be altered to due to changing environmental conditions such as drought, storms, or seasonal effects^59^. Antibiotic resistance and the ability of microorganisms to thrive in metal containing environments has been correlated with gene linkage^11,60^. Constructed wetlands have been found to be significant reservoirs of antibiotic and heavy metal resistance, that have been correlated with input content^61^. Spatial patterns in antibiotic resistance among stream bacteria have been shown to be correlated with heavy metal pollution at SRS^62^. Amendments such as sulfate have been applied without success to SRS wetland microcosms for enhanced Cu and mercury (Hg) removal by adsorption to organic matter, clays, and sulfate reducing microorganism for precipitation in anaerobic sediments^63^. Enhancement of aqueous copper removal in wetlands, constructed or otherwise, has been shown through the application of *Cupriavidus basilensis* SRS . However, to further enhance its applicability it may be possible to incorporate it into current industrial bioremediation processes such as commercially available exopolysaccharide biofilm producing consortia, which may stabilize the microbe. This biofilm formation could stabilize *Cupriavidus basilensis* SRS during inclement environmental factors such as fluxes in pH, conductivity, and other seasonal variations.

It is known that changing weather, storms, and temperature extremes can impact wetland sediments and associated metals. Seasonal biogeochemical cycling may also impact the fate of Cu and other metals in wetlands due to dynamic accumulation and metal redox cycling between macrophytes^64^. The H-02 wetland system has proven to be a significant resource not only for water treatment, but also for process control that can further enhance the engineered wetland function within the scientific and regulatory communities^6^. In this work we demonstrated that dissolved Cu could be significantly reduced in metal contaminated microcosms from the H-02 wetland through bioaugmentation. *Cupriavidus basilensis* SRS was isolated from the SRS making it a native species adapted to this location, giving it a competitive advantage. Further testing needs to be done to examine the stability of *Cupriavidus basilensis* SRS bioaugmentation under different environmental conditions to help predict the long-term fate and transport of microbially bound heavy metals. During the wetland microcosm testing the pH of the inoculated samples changed from 6 to 9 over 3 days which may suggest Cu^2+^ was transformed to Cu^+^^65^. This potential change could involve tenorite, a Cu oxide mineral with the chemical formula CuO illustrated in Fig. 7.

While the use of *Cupriavidus basilensis* SRS in the environment requires additional experimentation, it should be noted that the significant decrease in the aqueous concentration of Cu has considerable potential for both constructed wetlands designed for metal remediation and potentially other contaminated environments. For example, *Cupriavidus metallidurans* has shown potential to remediate mercury in polluted agricultural soil and various metal ions in media such as lead and cadmium^66^. Future work will include testing additional metals as well as the potential for inoculation of a larger sample size with *Cupriavidus basilensis* SRS . Additionally further work with the digestion of cell pellets to determine if the isolate can uptake Cu or if the Cu is binding to the sediment, as well as the speciation of the sorbed Cu. This information will contribute to useful remediation strategies for heavy metals that incorporate natural bioremediation in engineered systems. Furthermore, the application of natural bacteria could enhance the impact and lifetime of the constructed wetland and reduce toxicity and potential impacts on public health.
